# Five immune‐gene‐signatures participate in the development and pathogenesis of Kawasaki disease

**DOI:** 10.1002/iid3.373

**Published:** 2020-11-14

**Authors:** Han Nie, Shizhi Wang, Quanli Wu, Danni Xue, Weimin Zhou

**Affiliations:** ^1^ Department of Vascular Surgery The Second Affiliated Hospital of Nanchang University Nanchang Jiangxi China; ^2^ Medical College of Nanchang University Nanchang Jiangxi China

**Keywords:** immune‐related genes, Kawasaki disease, WGCNA

## Abstract

**Objective:**

To screen for immune genes that play a major role in Kawasaki disease and to investigate the pathogenesis of Kawasaki disease through bioinformatics analysis.

**Methods:**

Kawasaki disease‐related datasets GSE18606, GSE68004, and GSE73461 were downloaded from the Gene Expression Omnibus database. Three microarrays were integrated and standardized to include 173 Kawasaki disease samples and 101 normal samples. The samples were analyzed using CIBERSORT to obtain the infiltration of 22 immune cells and analyze the differential immune cells in the samples and correlations. The distribution of the samples was analyzed using principal component analysis (PCA). Immune‐related genes were downloaded, extracted from the screened samples and analyzed for differential analysis (different expression genes [DEG]) and weighted gene co‐expression network analysis (WGCNA). We constructed coexpression networks, and used the cytohobbe tool in Cytoscape to analyze the coexpression networks and select the immune genes that played a key role in them.

**Results:**

Immune cell infiltration analysis showed that B cells naive, T cells CD8, natural killer (NK) cells activated, and so forth were highly expressed in normal samples. T cells CD4 memory activated, monocytes, neutrophils, and so forth were highly expressed in Kawasaki disease samples. PCA results showed a significant difference in the distribution of normal and Kawasaki disease samples. From the screened samples, 97 upregulated and 103 downregulated immune‐related genes were extracted. WGCNA analysis of DEG yielded 10 gene modules, of which the three most relevant to Kawasaki disease were red, yellow, and gray modules. They were associated with cytokine regulation, T‐cell activation, presentation of T‐cell receptor signaling pathways, and NK cell‐mediated cytotoxicity. CXCL8, CCL5, CCR7, CXCR3, and CCR1 were identified as key genes by constructing a coexpression network.

**Conclusion:**

Our study shows that we can distinguish normal samples from Kawasaki disease samples based on the infiltration of immune cells, and that CXCL8, CCL5, CCR7, CXCR3, and CCR1 may play important roles in the development of Kawasaki disease.

## Graphical abstract

This study analyzed Kawasaki disease (KD)‐associated microarrays and found that normal and KD serum samples could be well distinguished according to the infiltration of immune cells, and then the key differential immune‐related genes were screened out. Immune‐related responses and play important roles, these genes may be key targets in the diagnosis and treatment of KD.

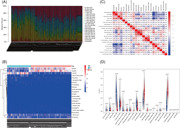

## 1 INTRODUCTION

Kawasaki disease (KD), also known as Kawasaki syndrome or mucocutaneous lymph node syndrome,^1^ is an acute febrile rash pediatric disease, majorly presenting with systemic vasculitis lesions. It is more prevalent in children under the age of 5 years and males but rare in adults and infants under 3 months old.^2^ Clinical manifestations of KD include fever with oropharyngeal changes, edema of the hands, and feet and enlarged lymph nodes.^3^, ^4^, ^5^ KD is more often acute (fever > 38.5°C) or subacute, where the latter develops from acute cases within just 2 weeks of no treatment. Studies show that KD often affects the small and medium‐sized arteries, particularly coronary arteries,^2^ therefore if untreated, it can lead to serious complications such as coronary aneurysm, thrombosis, stenosis, and even sudden death.^6^ In fact, up to 25% of patients with KD are at risk of severe coronary artery inflammation and aneurysm.^7^ Meanwhile, KD is currently one of the most common causes of acquired heart disease in children in developed countries.^8^ At the moment, KD is managed using high routine doses of intravenous immunoglobulin (IVIG), which reduces the risk of coronary aneurysms in these patients by 3%–6%.^9^, ^10^ Unfortunately, 10%–20% of patients with KD still experience persistent high fever and relapse after receiving IVIG.^11^ In addition, there is currently no gold standard for the diagnosis of the disease. Presently, diagnosis of KD is based on four out of five symptoms; fever for ≥5 days with strawberry tongue and cracked lips, conjunctivitis of both eyes, enlarged lymph nodes in the neck, edema of the extremities, and general rash.^12^ Ancillary examinations show elevated white‐blood cell and platelet count, increased C‐reactive protein, accelerated sedimentation of blood cells among others in KD.^12^ However, these clinical manifestations are not always apparent, which delays timely diagnosis and treatment of the disease.

Even though molecular biomedicine and in‐depth research have strengthened our understanding of KD, the main cause of the disease remains elusive.^13^, ^14^ In some quarters it is believed KD is a systemic vasculitis, whereas others believe it is a combination of infection and immune reaction triggered by specific pathogens attaching on the coronary arteries. KD is also thought to be an autoimmune disease arising from autoimmune dysregulation.^15^, ^16^, ^17^ Accordingly, immune genomics can give an insight into the molecular events leading to its development, in particular, immune cell infiltration. Findings of this study will create a foundation for the diagnosis and treatment of KD, as well as deepen our understanding on the etiology of the disease.

## 2 METHODS

### 2.1 Data acquisition and processing

GSE18606,^18^ GSE68004,^19^ and GSE73461^20^ immune gene expression datasets, comprising of 101 and 173 blood samples from the respective normal and KD individuals were downloaded from the Gene Expression Omnibus database (http://www.ncbi.nlm.nih.gov/geo/). The data were cleaned using Limma^21^ and SVA^22^ packages in R software (3.61).

### 2.2 Infiltration of immune cells in the samples

The immune cell expression profile between KD and normal tissues were analyzed using CIBERSORT tool,^23^ based on messenger RNA‐Seq data of the cells. Overall, we analyzed the relative abundance of 22 types of immune infiltrating cells, including natural killer (NK) cells, T cells, B cells, and macrophages. Statistical significance was set at *p* < .05.

### 2.3 Principal component analysis

Principal component analysis (PCA) was performed to realine the dimensionality of the 22 types of immune infiltrating cells, while still retaining key information.

### 2.4 Immune‐response‐related gene expression profiles

Expression profiles of 2498 genes related to immune infiltrating cells were downloaded from ImmPort (https://www.immport.org/home). The genes included those related to antigen‐presenting cells, chemokines and their receptors, cytokines and their receptors, interferons, and interleukins. Comparative expression of immune cells between normal individuals and those with KD was evaluated using the Limma package^21^ in the R software.

### 2.5 Screening of differentially expressed immune‐response‐related genes

After standardizing the data using Limma,^21^ we identified differentially expressed genes related to immune response during KD. Statistical significance for different expression genes (DEGs) was set at a change factor greater than onefold (|fold change| ≥ 1) and the corrected *p* value false discovery rate ≤ 0.05.

### 2.6 Construction of coexpression networks

Differentially expressed immune cells in 173 KD and 101 normal samples were analyzed using weighted gene co‐expression network analysis (WGCNA) package of R software (https://cran.rproject.org/web/packages/WGCNA/index.html), based on Pearson's correlation matrix; amn = |cmn|*β* (where amn is the closeness between gene m and n, cmn is the Pearson's correlation and *β* is the soft‐power threshold). WGCNA was majorly utilized in the construction of the gene coexpression network, identification and analysis of key disease‐associated modules, and construction of the protein network together with identification and enrichment analysis of key module hub nodes.

### 2.7 Gene Ontology and Kyoto Encyclopedia of Genes and Genomes enrichment analyses

Gene Ontology (GO) and Kyoto Encyclopedia of Genes and Genomes (KEGG) enrichment analyses were performed using clusterProfiler package in R software.^24^ Significance of differentially expressed genes in each signaling pathway was analyzed based on the hypergeometric distribution at *p* < .05.

### 2.8 Construction of coexpression networks of KD immune‐related genes

Key genes in coexpression networks were identified using WGCNA, and were then used to construct the protein–protein interaction (PPI) network. Meanwhile differential coexpression network of immune‐related genes was constructed using Cytoscape. The significance of coexpression was analyzed using the Cytohubbe tool.

## 3 RESULTS

### 3.1 Immune cell infiltration of samples

**Figure 1:**
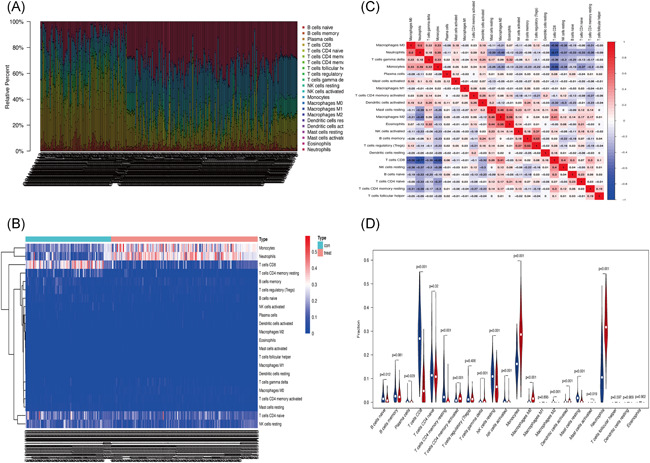
(A) Proportionate microarray analysis of the 22 immune cells in serum of individuals with or without Kawasaki disease. (B) Heat map for differential expression of immune cells in the samples. (C) Correlation between immune cells. (D) Immune cell infiltration (*p < .05, **p < .01, ***p < .001)

CIBERSORT analysis identified 173 KD and 101 normal samples exhibiting ideal immune cell infiltration (*p* < .05). As shown in Figure 1A, 22 immune cells infiltrated significantly different between the two sets of samples. In particular, monocytes, neutrophils, and T cells CD8 were the most significant differently expressed cells between the samples (Figure 1B). Correlation analysis revealed a strong negative correlation between CD8^+^ T cells and neutrophils, monocytes and M0 macrophages. On the other hand, eosinophils displayed a strong negative correlation with M2 macrophages, but positively correlated with M0 macrophages (Figure 1C). Figure 1D show 15 immune cells differentially expressed between normal samples and KD samples. Activated CD4^+^ memory T cells, gamma and delta T cells, monocytes, M0 macrophages, activated dendritic cells, activated mast cells, neutrophils, B cells naïve, plasma cells, CD8^+^ T cells, CD4^+^ resting memory T cells, resting and activated NK cells were all upregulated in KD. On the other hand, M2 macrophages and resting NK were overexpressed in normal samples.

### 3.2 PCA of the samples

**Figure 2:**
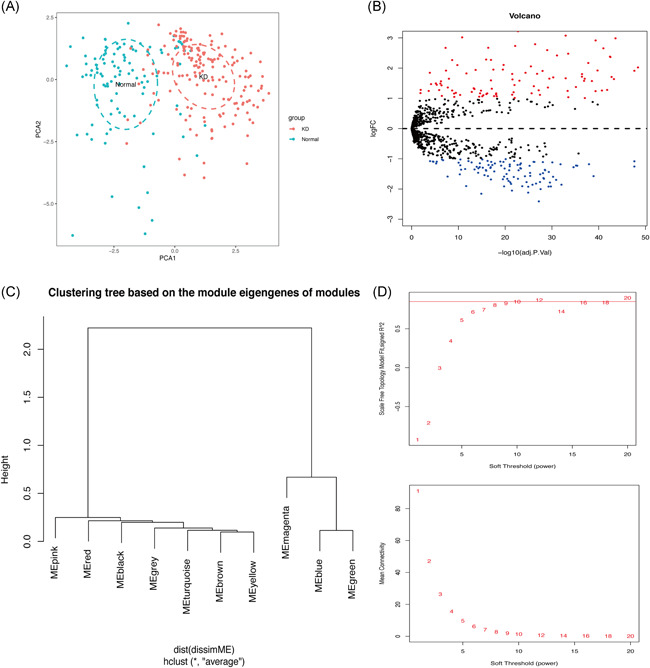
(A) Principal component analysis distribution of immune cells in normal and Kawasaki disease (KD) serum samples. (B) Volcano plot for differential expression of immune‐related genes in KD and normal tissues (blue dots represent downregulated genes whereas the red ones represents upregulated genes). (C) Outliers in the data were detected by clustering of samples. (D) Scale‐free fit indices obtained by soft threshold analysis of the topological network

PCA further validated the clear distinction between individuals with or without KD with regard to 22 immune infiltration cells (Figure 2A).

### 3.3 Screening of immune‐related genes

Analysis of immune‐related genes uncovered 97 and 103 genes that were respectively upregulated and downregulated between individuals with or without KD (Figure 2B).

### 3.4 Construction of gene coexpression network

**Figure 3:**
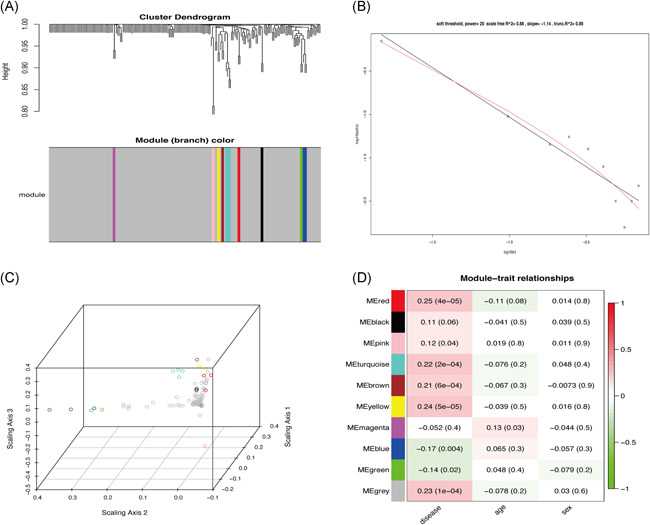
(A) Cluster analysis of the differentially expressed immune‐related genes data. (Each color represents a module in the gene coexpression network constructed by weighted gene co‐expression network analysis [WGCNA]). (B) Scale‐free fit index of 0.88 when the soft threshold *β* = 20. (C) Distribution of each WGCNA module. (D) The relationship between each module with disease, age, and sex

Differentially expressed immune‐related genes were identified using WGCNA (Figure 2C). Cells were clustered based on the *β* vaue, derived according to “sft$powerEstimate.” At *β* = 20, KD and normal tissues (Figure 2D) were classified into 10 modules (Figure 3A). At this threshold, the scale‐free topology was 0.88 (Figure 3B). The distribution of modules is shown in Figure 3C.

### 3.5 Module linkages and the relationship between WGCNA modules and clinical features

**Figure 4:**
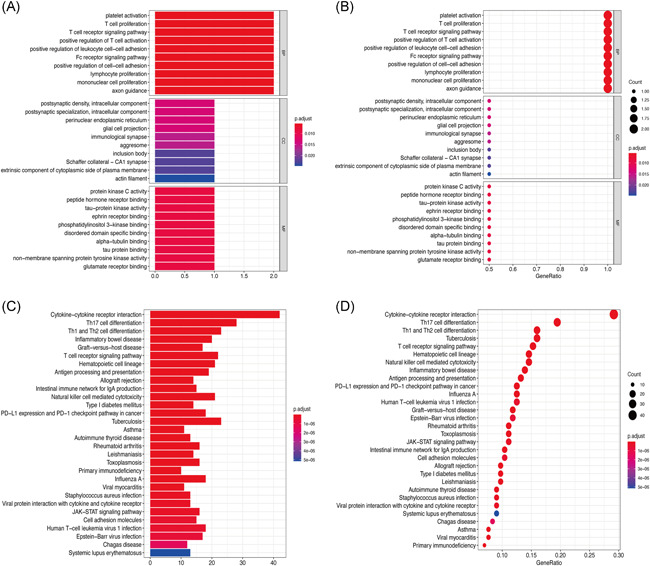
(A) Histogram for Gene Ontology (GO) enrichment analysis of red module genes. (B) GO enrichment analysis dot plot for red module genes. (C) GO enrichment analysis dot plot for gray module genes. (D) GO enrichment analysis dot plot for gray module genes

**Figure 5:**
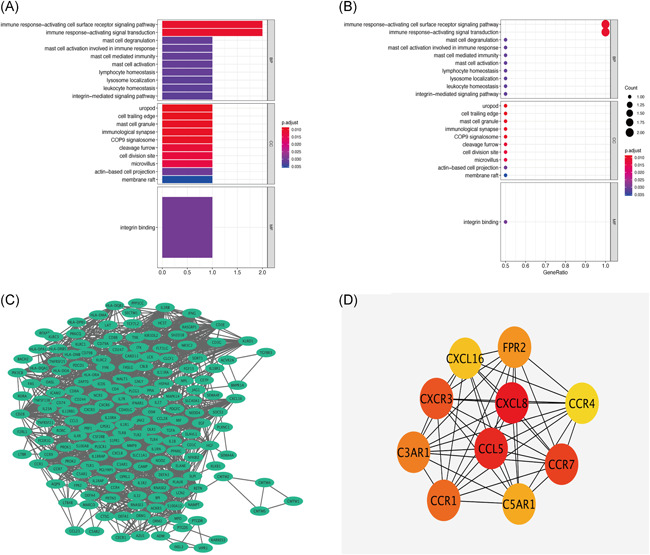
(A) Histogram for Gene Ontology (GO) enrichment analysis of yellow module genes. (B) Dot plot for GO enrichment analysis of yellow module genes. (C) Network construct of Kawasaki disease (KD) immune‐related genes. (D) Core immune‐related genes associated with KD (the darker the gene, the higher the score)

We constructed a graph depicting the relationship between different modules and clinical characteristics. KD was strongly associated with three modules; arbitrarily named red, yellow, and gray (Figure 3D). Functional enrichment analysis showed the red (Figure 4A,B) and gray modules (Figure 4C,D) were mainly associated with proliferation and activation of T lymphocytes. On the other hand, the yellow module was mainly associated with mast cell response (Figure 5A,B). Overall, connection analysis revealed an interlinkage among the three modules (Figure S1).

### 3.6 PPI networks

PPI network of the red, yellow, and gray modules (Figure 5C) identified CXCL8, CCL5, CCR7, CXCR3, and CCR1 as the key genes (Table 1) mediating the development and pathogenesis of KD. As shown in Figure 5D, the higher the degree value of the node, the darker the color and the larger the diameter.

**Table 1:** Key gene score

node_name	MCC	Type	Category
CXCL8	2.11E+13	Up	Antimicrobials
CCL5	2.11E+13	Down	Antimicrobials
CCR7	2.11E+13	Down	Antimicrobials
CXCR3	2.11E+13	Down	Chemokine_Receptors
CCR1	2.10E+13	Up	Cytokine_Receptors
C3AR1	2.09E+13	Up	Cytokine_Receptors
FPR2	2.09E+13	Up	Cytokine_Receptors
C5AR1	2.09E+13	Up	Chemokine_Receptors
CXCL16	2.09E+13	Up	Cytokines
CCR4	2.09E+13	Down	Cytokine_Receptors

Abbreviation: MCC, maximal clique centrality.

## 4 DISCUSSION

KD is an acute self‐limiting systemic vasculitis, first described by Kawasaki in 1967.^25^ Pathologically, KD affects coronary arteries, and at the moment, it is the most common cause of heart disease in children in developed countries.^26^ Unfortunately, the incidence of the disease is increasing in Japan.^27^ Though an old disease, the definite cause of KD is still unknown, 50 years after its discovery. The diagnosis of KD is confounded by in apparent clinical signs and symptoms. Accordingly, it is imperative to broaden our research into probable diagnosis approaches of KD, including profiling the expression of immune genes.

In this study, we analyzed the infiltration profile of 22 immune cells in 173 KD and 101 normal tissues based on 3 publicly available datasets. Monocytes, M0 macrophages, and neutrophils were significantly overexpressed in KD tissues. This immune profile was, in turn, associated with acute KD clinical symptoms such as periodic fever, lymphangitis, and oropharyngeal mucositis.^28^, ^29^, ^30^ Activated memory CD4+ T cells, delta, and gamma T cells also displayed a similar trend, consistent with findings of Abe et al.^31^, ^32^ On the other hand, the proportion of B cells naïve, T cells, CD8, and NK cells decreased significantly in KD. Although the underexpression of naïve B cells in KD has been demonstrated in several studies,^33^, ^34^, ^35^ the precise mechanism underlying this phenomena in the disease remains elusive. Expression profiles of T cells CD8 and NK cells are closely related to the progression of KD. Indeed Popper et al.^36^ demonstrated that T cells, CD8, and NK cells stick on the arterial walls or bind endothelial cells in acute phase of KD, but returned to normal circulation after recovery. Herein, PCA further demonstrated a clear distinction between KD samples and normal tissues (Figure 2A). Therefore, we performed WGCNA analysis, which stratified immune‐related genes into 10 modules. Three of them, arbitrarily named red, yellow, and gray were closely associated with KD. GO and KEGG analyses further revealed the three modules were associated with T‐cell responses, consistent with our previous immuno‐analysis. Network constructs of the red, yellow, and gray modules identified CXCL8, CCL5, CCR7, CXCR3, and CCR1 as the major immune genes involved in KD pathogenesis. One previous study in North India showed that mutations in CCL5‐403A genes are associated with coronary artery injury in children with KD.^37^ Meanwhile, expression of CCR7‐associated Treg cells peak in the acute and subacute phase of KD but declines during recovery. The self‐limiting nature of vascular inflammation during KD is, in fact, attributed to overexpression of Tregs.^38^ On the other hand, CXCR3, a G protein‐coupled receptor, is often associated with chemotaxis of immune cell and polarization of Th1 cells. Meanwhile, CXCL9, CXCL10, CXCL11 are the most important CXCR3 agonists. In a related study, Ko et al.^39^ showed that CXCL10 is significantly upregulated in acute KD, but is nonetheless, a positive prognostic factor of the disease. CXCL10, also a receptor for CXCR3, is also shown to be activated in the acute phase of KD. Independent studies have shown that the CXCL10/CXCR3 axis plays an extremely important immunomodulatory role in ischemic heart disease, myocarditis, leukoplakia, nonischemic heart failure, and KD, thus modulation of this axis is a potential immunotherapy target for these diseases.^40^ Although there are no studies on the role of CXCL8 and CCR1 in KD, these genes play important roles in immune‐related diseases such as vasculitis, Takayasu arteritis, glomerulonephritis, and granulomatosis with polyangiitis (Wegener's).^41^, ^42^, ^43^, ^44^ Moreover, CCR1 is prognostic factor in palliative inductive therapy for vasculitis.^45^ Taken together, we believe CXCL8, CCL5, CCR7, CXCR3, and CCR1 are the key immune‐related genes that participate in the development and pathogenesis of KD. Regarding limitations, this study lacked experimental validation. However, we shall undertake in‐depth follow‐up studies of the screened genes to establish the more precise mechanism underlying their involvement in the development and pathogenesis of KD.

## 5 CONCLUSION

CXCL8, CCL5, CCR7, CXCR3, and CCR1 are the major genes involved in the development and pathogenesis of KD. As such, they are potential targets in the diagnosis and treatment of the disease.

## CONFLICT OF INTERESTS

The authors declare that there are no conflict of interests.

## AUTHOR CONTRIBUTIONS

Han Nie: *Research design and drafting the manuscript*; Shizhi Wang: *Help to writing manuscript*; Quanli Wu: *Searching for references*; Danni Xue: *Reviewed the papers*; Weimin Zhou: *Review and revision of the manuscript and writing guidance*.

## Data Availability

The datasets used and/or analyzed during the current study are available from the corresponding author on reasonable request.
